# Person‐Centered Measurement: Ensuring Prioritization of Individuals’ Values, Needs, and Preferences Within the Global Contraceptive Measurement Ecosystem

**DOI:** 10.1111/sifp.70023

**Published:** 2025-06-23

**Authors:** Claire W. Rothschild, Kelsey Holt, Funmilola M. OlaOlorun, Julius Njogu, Abednego Musau, Christine Dehlendorf

## Abstract

Despite growing enthusiasm for measurement approaches that capture individuals’ needs, values, and preferences, there remains a lack of conceptual clarity regarding person‐centered measurement in the global field of contraception. In this commentary, we propose a working definition of person‐centered measurement within the contraceptive field and describe how this definition can be applied to existing and novel contraceptive indicators. We argue that person‐centered measures of contraception must both reflect an individual's self‐identified values, needs, and/or preferences related to contraception *and* allow an individual to assess the extent to which these values, needs, and/or preferences have been fulfilled. As a result, a person‐centered measure allows the individual to define for themselves whether a “good” outcome has been achieved. While person‐centered measures are a critical component of measuring the performance of contraceptive programs, measurement frameworks must also include non‐person‐centered measures that allow evaluation of normative constructs such as human rights and reproductive justice.

## INTRODUCTION

There is a growing consensus in the global field of contraception that new measures and measurement approaches are needed (Speizer, Bremner, and Farid 2022; Fabic 2022; Senderowicz 2020). Critiques of standard indicators used to monitor and evaluate contraceptive programs—many of which were developed during the height of the population control era and have remained little‐changed in the intervening half century (Bradley and Casterline 2014)—highlight the problematic approaches of defining contraceptive use as a universally positive outcome and of classifying contraceptive “need” for women without directly asking them what they need or want (Speizer, Bremner, and Farid 2022; Rothschild, Brown, and Drake 2021; Holt, Galavotti, et al. 2023; Rothschild et al. 2024; Senderowicz 2020). Take, for example, the core indicators used by the Family Planning 2030 (FP2030) initiative to track global progress in family planning (FP) programming (Online Appendix: Supplemental Materials, Table A1): Nine of the 22 indicators measure contraceptive use, focusing on modern methods; three indicators capture aspects of supply‐side contraceptive method availability; and another six indicators measure fertility and health outcomes, such as number and percentage of births that are unintended (Track20). Only one indicator measures quality of contraceptive counseling, while another single indicator captures self/joint contraceptive decision‐making—an aspect of contraceptive autonomy (Senderowicz 2020). FP2030 has itself highlighted the need for “improving measurement of rights and empowerment principles” (FP2030 2023). Similar critiques have been leveled at the U.S.’s Healthy People 2030 public health initiative, which includes a performance indicator on the use of effective birth control methods (Gomez et al. 2024).

In March 2024, an international expert working group meeting was convened by the International Union for the Scientific Study of Population (IUSSP) in Mombasa, Kenya, under the auspices of “Rethinking Family Planning Measurement with a Reproductive Justice and Rights Lens” (IUSSP 2024). A focus of the expert group meeting and following convenings was to align on a global measurement “ecosystem”—or set of priority indicators—that better reflects principles of human rights and reproductive justice in FP programming. To do so, the group discussed the need for inclusion of indicators related to contraceptive agency, self‐efficacy, and autonomy, and social and gender norms relevant to contraception and reproductive health more broadly. In addition, the group discussed the need for measures that explicitly capture people's individual preferences and subjective experiences. While much of the conversation focused on the need for so‐called “person‐centered” measures, several discussions during the meeting of what person‐centeredness means as applied to measurement and how it in turn relates to principles of human rights and reproductive justice went unresolved. Notably, there was a lack of common understanding of whether measures of agency, empowerment, or one's intention to use contraception are inherently person‐centered (IUSSP 2024). The goal of this commentary is to improve conceptual clarity on person‐centeredness in the context of contraception, focusing on the role of person‐centered measures in advancing a broader rights‐based and justice‐informed contraceptive measurement agenda.

## WHAT MAKES A CONTRACEPTION‐RELATED MEASURE OR INDICATOR “PERSON‐CENTERED”?

In its landmark 2001 report, the U.S. Institute of Medicine (IOM) Committee on Quality of Health Care brought this terminology to the global health stage. The IOM defined patient‐centered care as “care that is respectful of and responsive to individual patient preferences, needs, and values and [that ensures] that patient values guide all clinical decisions” (IOM 2001). Following the IOM report, there were calls for the need to expand focus from the “patient”—the narrowly defined and passive beneficiary of care—to a holistic “person” with multifaceted values and experiences that influence their healthcare preferences and outcomes. This critique served as the basis for a reconceptualization of *patient*‐centered care as *person*‐centered care (Ekman et al. 2011). Reflecting the fundamental concept of patient‐centered care, person‐centered care aims to put people at the center of their health care through approaches such as empathy, respect, shared decision‐making, and personalization (Eklund et al. 2019).

It follows that a contraception‐related measure can be defined as person‐centered if it assesses to what extent the construct being studied aligns with what people *themselves* want, need, and/or value related to contraception. Yet, myriad examples exist in the contraception literature of a broader interpretation of the concept of person‐centeredness as it relates to measurement. An example of the lack of standardized terminology related to person‐centered measurement is illustrated by a recent initiative aiming to measure person‐centered contraceptive care, which describes a “person‐centered measurement” approach that uses simulated clients, reflections with clinicians involved in implementing the intervention, and comparative analysis of healthcare providers’ language (Baayd et al. 2024). While simulated clients, clinician interviews, and analysis of provider language can be used to measure person‐centered *care*, these measurement approaches themselves are not person‐centered because they produce measures that do not capture clients’ own assessments of the care received.

We propose that person‐centered *measurement* is fundamentally different from the measurement of person‐centered *care* itself: the former is related to how the measure is framed and interpreted, while the latter is related to the construct being measured—for example, content or processes of care that are person‐centered. In their 2018 narrative review of interventions to improve person‐centered quality of FP care, Diamond‐Smith, Warnock, and Sudhinaraset make a distinction between “person‐centered care *processes* (i.e., dignity, autonomy, privacy/confidentiality, communication with providers/patients, social support in the facility including family members, supportive care, and trust in providers) and person‐centered *outcomes* (i.e., patient satisfaction and experiences)” (Diamond‐Smith, Warnock, and Sudhinaraset 2018). In the example above, we argue that methods to assess person‐centered care through means that are not self‐reported (such as simulated clients or clinician interviews) are valid methodologies for assessing person‐centered care processes. However, they are not person‐centered measurement approaches: first, they do not capture individual's self‐identified needs, values, and/or preferences; and second, they do not assess the extent to which the care provided aligns with those needs, values, and/or preferences. Sudhinaraset's Person‐Centered Family Planning scale provides another example of a measure of person‐centered care that is not itself person‐centered. To assess the extent to which care is dignified and respectful, for example, the scale includes a question about whether the healthcare provider called the client by their name (Sudhinaraset et al. 2018). Because this item is interpreted based on a normative, universal standard for person‐centered care (e.g., calling the client by their name is “good” person‐centered care), it does not reflect an individual's values and preferences and therefore is not—as a measure—person‐centered.

Coming back to the idea of a measurement “ecosystem” for the contraception field, we emphasize that not all measures in the ecosystem *should* be person‐centered. Person‐centered measures are just one component of assessing fulfillment of rights and reproductive justice; a measurement ecosystem would not be comprehensive if it did not also include “objective” indicators, such as those related to technical quality of care. Person‐centered measures reflect an individual's subjective experiences. An individual's assessment of quality of care, for example, may demonstrate that the care they received fully met their expectations, even when care fails to meet a minimum threshold for technical quality (Thompson and Sunol 1995; Bjertnaes, Sjetne, and Iversen 2012). For this reason, technical measures of care quality that are not person‐centered are often critical for the appropriate interpretation and actioning of patient satisfaction indicators.

## PROPOSED DEFINITION OF A PERSON‐CENTERED CONTRACEPTION MEASURE

To provide clarity around the meaning of person‐centeredness in contraception measurement, we propose a definition that hews more closely to definitions of patient‐ and person‐centeredness in the health literature (Eklund et al. 2019; IOM 2001; Diamond‐Smith, Warnock, and Sudhinaraset 2018).

We suggest that a measure (or indicator) can be considered person‐centered if it meets both of the following criteria:

1. *The measure assesses an individual's* self‐identified *values, needs, or preferences*: Because person‐centeredness centers the individual, a person‐centered measure must capture that individual's self‐identified values, needs, and/or preferences. A person‐centered measure cannot be one that is directly observed by the researcher (e.g., an observation of the client–provider interaction) or reported by the provider (e.g., a provider knowledge test) because such external assessments lack consideration of the individuals’ own perspectives or experiences. Even if a measure is reported by the individual (e.g., self‐ or person‐reported), it may not meet this requirement *if* it does not capture some aspect of the individual's values, needs, and/or preferences.
2. *The measure reflects the individual's own definition of a “good” or a “bad” outcome*: In order to reflect the degree of person‐centeredness of a particular outcome, person‐centered measures must capture the extent to which an individual's experiences or behaviors align with or fulfill their own self‐defined values, needs, and/or preferences. Thus, a person‐centered measure must reflect the person's own assessment of the extent to which their needs, values, and preferences have or have not been met. A person‐centered measure is one that allows the individual—rather than the researcher or an external normative framework—to define what a “good” versus a “bad” outcome is for themselves related to the construct being measured.

Take, for example, two common indicators used in contraceptive research: intention‐to‐use contraception in the future and fertility intentions (e.g., desire for a [or another] child in the future). Both indicators meet criterion #1, as they are reported by the individual under study and they reflect that individual's self‐identified preferences for future contraceptive use and future fertility, respectively. We would not consider either measure to be person‐centered, however, because neither indicator reflects the individual's assessment of the extent to which their preferences have or have not been met (criterion #2), thus leaving interpretation of the measure as “good” or “bad” open to the observer, researcher, or other consumers of the data.

It is important to note that criterion #2 (above) can be satisfied in two ways. First, individuals can be asked to directly assess the extent to which a construct (e.g., behavior or experience) aligns with their preferences, values, and/or needs. For example, Bullington et al. (2023) have described “self‐perceived” measures of informed choice in contraceptive counseling, which directly ask the respondent whether they received enough information to make a good choice for themselves about contraception. In this example, the respondent assesses for themselves what “enough information” means based on their own values and preferences, thereby fulfilling both criteria above and making this a person‐centered measure. This can be contrasted with what Bullington et al. call “researcher‐ascribed” measures of informed contraceptive choice, in which the researcher makes normative judgments about requirements that must be met in order for informed choice to occur.

In addition to person‐centered measures that directly ask participants to evaluate the extent to which an experience or behavior aligns with their preferences, there is a second, emerging type of person‐centered indicator that indirectly assesses to what extent the construct being measured is concordant (or discordant) with individual preference. Holt, Galavotti, et al. (2023) have proposed, for example, a novel measure called preference‐aligned fertility management (PFM), which is constructed by asking the respondent if they want to be using contraception right now; if they *are* using contraception right now; and if the contraceptive method they are using right now is the one they want to be using. A respondent is coded as practicing PFM (a “good” outcome) if their contraceptive use and method type align with their preferences, irrespective of whether their preference is to use or not use contraception. Another indicator of this type proposed by Burke and Potter is preferred method use, which is defined as concordance between use and stated preference for a specific contraceptive method type (including non‐use as an acceptable preference) (Burke and Potter 2023; Gomez et al. 2024). The PFM and preferred method use indicators differ from the “self‐perceived” measures described previously in that they do not directly ask the individual whether her contraceptive use matches her preferences. However, because PFM is interpreted based on the respondent's self‐identified preferences—what the respondent says she wants—rather than based on some external normative framework, we consider PFM and preferred method use to fulfill both criteria above and to be person‐centered measures.

While straightforward in theory, defining what does and does not qualify as a person‐centered measure becomes more complicated upon application. Table 1 presents several examples of measures of the same contraceptive constructs that we would and would not consider to be person‐centered measures. Below, we reflect on several areas of possible confusion.

**TABLE 1:** Illustrative examples of person‐centered and *non*‐person‐centered measures of contraceptive constructs

Construct	Example of a person‐centered measure	Analogous measure that is *not* person‐centered
Dignified/respectful contraceptive care	*Item from Quality of Contraceptive Counseling Short Version (QCC‐10) scale* (*Holt, Karp, et al.* 2023): The provider gave me the time I needed to consider the contraceptive options we discussed	*Item from Performance Monitoring for Action (PMA) client exit interview questionnaire*: How long did you wait between the time you arrived at this facility and the time you were able to see a provider for the consultation (in minutes/hours)? *(Does not fulfill criteria #1 or #2)*
	*Item from the Person‐Centered Family Planning Scale (* *Sudhinaraset et al.* 2018 *)*: Did the doctors, nurses, or other staff at the facility treat you with respect?	*Item from EngenderHealth REDI Client‐Centered Counseling Skills Observation Checklist (EngenderHealth)*: Did the provider greet the client with respect? *(Does not fulfill criteria #1 or #2)*
Informed contraceptive choice	*Item from the PCCC measure (* *Dehlendorf et al.* 2021 *)*: Think about your visit. How do you think [Provider Name] did: Giving me enough information to make the best decision about my birth control method.	*Method Information Index (* *Jain et al.* 2019 *)*: 1. Were you informed about other methods of family planning? 2. Were you informed about possible side effects or problems you might have with the method? 3. Were you told what to do if you experience any side effects or problems? *(Does not fulfill criteria #1 or #2)*
	*Item from the QCC‐10 scale (* *Karp et al.* 2023 *)*: I received all the information I wanted to know about my options for contraceptive methods.	*Contraceptive Autonomy Scale ‐ Informed Choice Domain (* *Senderowicz et al.* 2023; *Senderowicz* 2020) 1. Knows how to use a method from each group 2. Knows a benefit/advantage of non‐use of family planning 3. Knows a risk/disadvantage of non‐use of family planning 4. Knows a benefit/advantage of their method 5. Knows a risk/disadvantage of their method 6. Knows what to do in case of side effects 7. Was told about the method of removal/permanence *(Does not fulfill criteria #1 or #2)*
Contraceptive use	*Preference‐aligned fertility management index (* *Holt, Galavotti, et al.* 2023 *)*: Concordance between contraceptive use and method type and one's preferences, irrespective of whether their preference is to use or not use contraception	*Unmet need for family planning measures*: Discordance between contraceptive use and stated fertility intentions *(Fulfills criterion #1 but does not fulfill criterion #2)*
		*Intention‐to‐use contraception measure*: Stated intention to use contraception or not in the future *(Fulfills criterion #1 but does not fulfill criterion #2)*

## HOW DO PERSON‐CENTERED MEASURES DIFFER FROM RIGHTS‐BASED OR REPRODUCTIVE JUSTICE‐INFORMED MEASURES?

**FIGURE 1:**
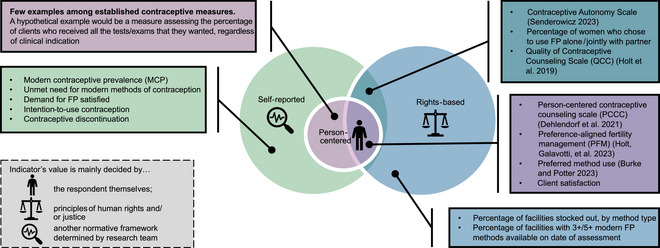
A classification of illustrative person‐centered, rights‐based, and/or self‐reported family planning indicators

A human rights‐based approach is one that is “normatively based on international human rights standards and operationally directed to promoting and protecting human rights” (United Nations 2024). Extending this definition to measurement, a rights‐based measure's purpose is to assess to what extent human rights are being fulfilled, regardless of whether an individual themselves expects or demands those rights. The Reproductive Justice framework, developed by Black American feminists following the 1994 International Conference on Population and Development, is itself a rights‐based framework that builds upon principles of human rights and emphasizes the role that intersectional forms of oppression—including racism, classism, and sexism—play in determining whether individuals’ human right to reproductive autonomy is upheld (Gilliam, Neustadt, and Gordon 2009; Cadena, Chaudhri, and Scott 2022; In Our Own Voice: National Black Women's Reproductive Justice Agenda 2024). Rights‐based measures, including reproductive justice‐informed measures, are inherently normative, in that their design and interpretation are based on a conceptual model that defines whether an outcome is a positive one or not according to whether an individual's right to reproductive freedom is upheld. Given that an individual's own preferences may differ from what is defined as positive according to a rights‐based lens, we argue that rights‐based measures related to contraceptive programming are not inherently person‐centered (Figure 1). Critical to this distinction is that the main decision‐maker—the actor or set of principles that guide how the measure is interpreted—differs by measure type: for a person‐centered measure, the main decision‐maker is the individual themselves, whereas for a rights‐based (but non‐person‐centered) measure, this decision is guided by a set of principles (e.g., human rights or reproductive justice).

Examples of rights‐based contraceptive measures include but are not limited to measures related to availability and technical quality of contraceptive care—for example, measures of healthcare providers’ clinical knowledge; availability of a specific number of different contraceptive method types within a facility; and contraceptive method stock outs. These measures can be considered rights‐based in that they aim to assess whether care was available and technically appropriate; they are not person‐centered measures, because interpretation is guided by a normative, rights‐based conceptual model of what high‐quality contraceptive care should include and not by the person's own perception of the adequacy or appropriateness of the care they received. To provide another example, Sustainable Development Goal indicator 5.3.1 on early marriage (the proportion of girls and women aged 20–24 years who were married or in union before age 15 and before age 18) is considered a rights‐based indicator reflective of gender equality and empowerment. We do not consider this indicator to be person‐centered, as it does not capture the individual's perspectives, values, or preferences related to early marriage. Similarly, the percentage of women who report deciding to use FP alone or jointly with their partner is a Demographic and Health Survey indicator and a core indicator in the FP2030 measurement framework, widely interpreted as a reflection of individual autonomy and voluntariness of FP use (FP2030 2023). Self/joint FP decision‐making is a justice‐informed measure that is *not* person‐centered under our definition, as interpretation is based on normative values of autonomy and not on whether the respondent's FP decision‐making aligns with her individual preferences and values.

For another example, we take the case of measurement of informed contraceptive choice. Using a justice‐informed conceptual model of informed contraceptive choice, Senderowicz et al. (2023) have proposed that informed choice occurs when a number of factors are in place, including knowledge of a range of different contraceptive methods; understanding of both advantages and disadvantages of the selected method; and knowledge of side effects of the selected method, among others. Scoring of the informed choice domain of Senderowicz's Contraceptive Autonomy Scale—to what extent informed choice is present or not—is guided by whether all items comprising informed choice are present, rather than by the respondent's own assessment (Senderowicz 2019, 2020). This approach can be contrasted with the informed choice‐related item of the Person‐Centered Contraceptive Counseling (PCCC) measure, which asks contraceptive clients, “How do you think your provider did giving [you] enough information to make the best decision about [your] birth control method?” (Dehlendorf et al. 2021). In this example, the respondent assesses for themselves what “enough information” means based on their own values and preferences, making this a person‐centered measure. It is important to note that a limitation of some person‐centered contraception measures is that they assume that the construct being measured, such as information or choice, is in itself valued by the respondent. For example, in the case of the PCCC measure, the respondent defines for themselves whether informed choice occurred, yet they are not asked the degree to which they value informed contraceptive choice in the first place.

Defining rights‐ and justice‐based measures as distinct from person‐centered measures does not indicate that one type of measure is preferable. Rather, both are important and reflect different aspects of the extent to which programs and policies are enabling optimal contraceptive experiences. For instance, in the example of the PCCC and the Contraceptive Autonomy Scale measures, both capture important but distinct aspects of informed contraceptive choice. The former is focused on whether people are receiving care that meets their needs, given their current context and desires, which is critical for understanding people's experiences in their sociocultural context. In contrast, the latter provides insight into whether care is aligned with human rights principles.

## ARE ALL MEASURES OF CONTRACEPTIVE EMPOWERMENT NECESSARILY PERSON‐CENTERED?

Because the constructs of self‐efficacy, agency, autonomy, and empowerment center respect of individuals, it may be falsely assumed that measures of these constructs are person‐centered by definition. As we argued in the case of person‐centered outcomes versus person‐centered processes, measures of person‐centered constructs are not necessarily themselves person‐centered. The Monitoring and Evaluation to Assess and Use Results (MEASURE) Evaluation's Reproductive Empowerment Scale is an example of a measure of empowerment that we would not define as person‐centered. In the MEASURE scale, participants who respond affirmatively to statements such as “you can initiate conversations about using contraception with your partner” are coded as having higher levels of empowerment than respondents who respond in the negative (Mandal and Albert 2020). While covert use is often driven by inequitable gender norms and patriarchal social and familial structures (Kibira et al. 2020), qualitative studies highlight the nuances of covert use, with some covert users expressing views that male partners should not be involved in contraceptive decisions and others viewing covert use as an example of empowered decision‐making within a constrained context (Kibira et al. 2020; Hoyt et al. 2022). If such a measure were redesigned from a person‐centered perspective, it might ask women the extent to which they could exercise power in their decisions to use contraception in the way that they want to—thereby leaving interpretation of power to the individual. Further, as is increasingly recognized within the contraception field, one's right to informed choice related to contraception is contingent upon the individual's agency in decision‐making—not just related to having information but their ability to form values‐based preferences and have critical consciousness of the societal conditions that constrain their reproductive autonomy (Holt et al. 2024; Edmeades et al. 2018). Emergent measures of agency related to contraceptive decision‐making will fill an important void within the measurement ecosystem but will not all inherently be *person‐centered*; for example, measures of critical consciousness capture empowerment *processes* but are not person‐centered outcomes as individuals’ own preferences and values are not considered.

## HOW DO PERSON‐CENTERED MEASURES DIFFER FROM PATIENT‐REPORTED OUTCOMES?

Health services researchers have increasingly called for the use of patient‐reported outcomes (PROs) in assessing person‐centered care and healthcare quality (Garcia and Spertus 2021; Liu, Bozic, and Teisberg 2017; cms.gov Measures Management System 2023). In the context of value‐based payments in the U.S. healthcare landscape, Liu, Bozic, and Teisberg (2017) propose that person‐centered measurement should focus on measures of healthcare quality that reflect patients’ stated priorities for care and their experiences of care. Under our definition, all person‐centered measures would be defined as PROs. However, while some PROs qualify as person‐centered measures under this definition, many do not: being self‐reported is necessary for meeting criterion #1 for our proposed definition of a person‐centered measure but not sufficient. To fulfill criterion #1, a measure must be both self‐reported *and* the measure must capture some aspect of preference, values, or self‐identified needs. Take as an example patient‐reported measures of physical activity. Such measures meet the definition of PROs (since they are self‐reported by the patient) but would not be considered person‐centered under our definition, because self‐reported physical activity measures do not capture individual values, preferences or needs (criterion #1) or the individual's interpretations regarding what level/type of physical activity is “good” versus “bad” according to their own values (criterion #2).

## CONCLUSION

In this commentary, we attempt to address some of the unresolved conceptual discussions at the 2024 IUSSP panel related to how person‐centered measurement fits within the movement towards a global rights‐based and justice‐informed measurement ecosystem—as described above, a suite of measures that, together, provide a comprehensive picture of the status of contraceptive experiences from multiple perspectives within a particular program, country, region, or globally (IUSSP 2024). We put forth a definition for person‐centered contraception measures based on two criteria: person‐centered measures are (1) self‐reported and capture constructs related to values, preferences, and/or needs and (2) with response scales that reflect the individual's own interpretation of what is a “good” or a “bad” outcome. As the global contraceptive community continues to critique, develop, and validate new measures, we emphasize that person‐centered measures are one (but certainly not the only) important component of a measurement ecosystem. Normative measures and measurement approaches will play a critical role in such a framework: to hold contraceptive programming accountable to a set of universal standards rooted in human rights and reproductive justice frameworks. Indeed, in order to comprehensively assess whether contraceptive programming is meeting rights‐based and justice‐informed standards, we need to include *non*‐person‐centered indicators, including measures of the health system and policy environment; technical quality of care; and the extent to which care incorporates essential person‐centered processes to uphold dignity, respect, and autonomy. However, it is also critical that person‐centered measures that capture people's lived experiences and subjectivities, which are not currently included in the suite of global sexual and reproductive health and rights (SRHR) measurement frameworks (FP2030 2023), be prioritized. Qualitative data will be critical to the development and validation of such measures, ensuring that quantitative, person‐centered measures validly reflect individuals’ own qualitative assessments of the construct under study. The inclusion of such qualitatively validated, person‐centered measures within a suite of contraceptive indicators will ensure that global efforts to measure progress in global contraceptive programming capture not only universal standards, but also reflect the needs, values, and preferences of the individuals whom these global efforts are meant to serve.

## CONFLICT OF INTEREST STATEMENT

The authors have no conflicts of interest to disclose.

## Supporting information



Appendix
