# Supplementary material for: Person‐Centered Measurement: Ensuring Prioritization of Individuals’ Values, Needs, and Preferences Within the Global Contraceptive Measurement Ecosystem
Source: Stud Fam Plann. 2025 Jun 23;56(3):390–402. doi: 10.1111/sifp.70023 (PMC12501692; doi:10.1111/sifp.70023)
Supplement: Supplementary file 1 — Appendix [file SIFP-56-390-s001.docx]

**Appendix: Supplemental Materials**

**Table A1. FP2030 core indicators, by conceptual domain**

| **Indicator** | **Conceptual Domain** |
| --- | --- |
| Modern contraceptive prevalence | Contraceptive use |
| Percentage of women estimated to have an unmet need for modern methods of contraception | Contraceptive use |
| Percentage of women estimate to have their demand for family planning met with a modern method of contraception | Contraceptive use |
| Total number of users of modern contraceptive methods | Contraceptive use |
| Contraceptive method mix | Contraceptive use |
| Contraceptive discontinuation rates | Contraceptive use |
| Contraceptive method switching | Contraceptive use |
| Method Information Index Plus | Quality of care |
| Percentage of women who had interactions with the health care system and among those women, the percentage that received FP information during contact with a health service provider | Access |
| Percentage of facilities stocked out, by method offered, on the day of assessment | Access |
| Percentage of primary SDPs that have at least 3 modern methods of contraception available on day of assessment | Access |
| Percentage of secondary/tertiary SDPs with at least 5 modern methods of contraception available on day of assessment | Access |
| Couple-years of protection (CYPs) | Contraceptive use |
| Percent of current modern contraceptive users who last obtained their family planning method from each source | Contraceptive use |
| Annual expenditure on family planning from government domestic budget | Political commitment |
| Adolescent birth rate | Fertility and health outcomes |
| Number of unintended pregnancies | Fertility and health outcomes |
| Percent of births that are unintended | Fertility and health outcomes |
| Number of unintended pregnancies averted due to modern contraceptive use | Fertility and health outcomes |
| Number of unsafe abortions averted due to modern contraceptive use | Fertility and health outcomes |
| Number of maternal deaths averted due to modern contraceptive use | Fertility and health outcomes |
| Percentage of women who decided to use family planning alone or jointly with their husbands/partners | Contraceptive autonomy |
